# Evaluation of Drug-Related Receptors in Children With Dilated Cardiomyopathy

**DOI:** 10.3389/fped.2019.00387

**Published:** 2019-10-01

**Authors:** Qing Guo, Jie Liu, Peng Zhu, Yali Liu, Nianguo Dong, Jiawei Shi, Hua Peng

**Affiliations:** ^1^Department of Pediatrics, Union Hospital, Tongji Medical College, Huazhong University of Science and Technology, Wuhan, China; ^2^Department of Cardiovascular Surgery, Union Hospital, Tongji Medical College, Huazhong University of Science and Technology, Wuhan, China

**Keywords:** children, dilated cardiomyopathy, receptors, treatment, RAAS

## Abstract

**Background:** Effective treatments for pediatric dilated cardiomyopathy (DCM) are limited. Currently, pediatric DCM therapy mainly includes supportive heart failure (HF) treatment. While the treatment for child DCM patients is generally the same as that for adult DCM patients, few randomized prospective studies on the clinical efficacy of treatments for pediatric DCM have been published. We explored the appropriate treatments for child patients.

**Methods:** The ultrastructure of pediatric DCM and control hearts was analyzed by electron microscopy and HE staining. Left ventricular tissues from children in the DCM and control groups were subjected to quantitative RT-PCR (qRT-PCR) to study the mRNA expression of receptors related to various treatments, including drugs targeting the renin-angiotensin-aldosterone system (RAAS) system, digoxin, milrinone, and β-receptor blockers, in child patients in the clinic. Furthermore, the differences in drug receptors in heart tissues between children and adults with DCM were analyzed.

**Results:** Compared with the control children, the children in the DCM group showed marked abnormalities in structure and organelles. The mRNA levels of angiotensin-converting enzyme (ACE), REN, prorenin receptor (PRR), NEP, ATP1A1, and phosphodiesterase3 (PDE3A) were higher in the pediatric DCM group than the control group. Interestingly, the mRNA expression of these treatment-related receptors was much higher in children than in adults.

**Conclusion:** ACE inhibitors, PRR or REN receptor inhibitors, PDE3 inhibitors and LCZ696 may be effective in children with DCM. However, β-receptor blockers are not valid treatments for pediatric DCM. Moreover, high receptor expression was observed in children. These data will improve the selection of drugs for DCM patients, enhance treatment, and increase the survival rate.

## Introduction

Dilated cardiomyopathy (DCM) is a kind of cardiomyopathy that involves left ventricular (LV) dilation and systolic dysfunction without abnormal load conditions and severe coronary artery disease (1). The annual incidence of pediatric DCM is 0.58 cases per 100,000 person-years (2). Effective treatments for pediatric DCM are limited. Currently, therapy for DCM in children mainly includes supportive heart failure (HF) treatment. According to the current guidelines, pharmacotherapy involving ß-blockers and angiotensin-converting enzyme (ACE) inhibitors is the basis of pediatric HF therapy; however, viable randomized prospective studies are lacking (3). In addition, treatments such as furosemide, milrinone, and digoxin, which are commonly used to treat adult HF, are used to treat child HF. Although, the current treatment of adult HF has shown some success with these drugs, these same therapies do not seem to be effective for pediatric HF. Specifically, treatment of adult HF with PDE3A inhibitors led to an increased incidence of sudden death and arrhythmias (4). Treatment with PDE3A inhibitors improved symptoms in pediatric HF patients without increasing the incidence of arrhythmias or sudden death (5).

Activation of β-adrenergic and renin-angiotensin-aldosterone system (RAAS) receptors plays an important role in the treatment of adult HF and cardiac remodeling (6). RAAS is important regulator of blood pressure, and suppression of RAAS can decrease cardiac load and relieve HF. RAAS include some classic receptors such as NR3C2, prorenin receptor (PRR), ACE, REN. The β-adrenergic receptors of the myocardium play an important role in the regulation of heart function. Many studies reported that an over-expression of the ADRB1 (β1-adrenoceptor) are which is one of the main β-adrenergic receptors may contribute to heart failure. Treatment of HF and atrial fibrillation with cardiac glycosides can inhibit the Na+, K+-ATPase. A large catalytic subunit (alpha) and a small glycoprotein subunit (beta) compose Na+, K+-ATPase. The ATP1A1 gene encodes an alpha 1 subunit with good affinity for digoxin (7). These genes are involved in HF and LV remodeling (8). A member of the cGMP-inhibited cyclic nucleotide phosphodiesterase family is encoded by the PDE3A gene. Thus, inhibition of the protein encoded by the PDE3A gene may be an effective treatment for congestive HF (9). Neprilysin encoded by the NEP gene is a neutral endopeptidase that is a potential target for the treatment of HF and hypertension due to its functions in vasodilation, natriuresis, and fibrosis (10).

We suggest that the mechanisms of HF may be different in children and adults and that different cellular mechanisms may be involved in pediatric HF. We evaluated the effect of drugs on the treatment of children with DCM by measuring mRNA expression of the corresponding drug-related receptors in cardiomyocytes after HF treatment in this study.

## Materials and Methods

### Human Samples

Child DCM samples (*n* = 11; age < 16 years) were obtained from Wuhan Union Hospital from January 2017 to October 2018 during heart transplantation due to end-stage idiopathic DCM. All cases of children DCM who underwent heart transplantation in this study were confirmed as primary DCM through discussion and approval by members of the heart transplantation committee. In addition, this research ethics has been approved by the medical ethics committee of Tongji medical college, Huazhong University of Science and Technology and all the patients' family members had signed informed consent before taking samples of this study. The clinical history and blood tests of these pediatric patients were available (Table 1). Adult DCM samples (*n* = 10; age 20–60 years) were obtained from Wuhan Union Hospital from January 2016 to 2018 from patients who underwent transplantation due to end-stage DCM and had no cardiac complications, such as hypertension, coronary atherosclerosis, and myocarditis. Control samples (*n* = 7) were from donor hearts that could not be transplanted for technical reasons (blood type or size mismatch) with normal LV function and active infection or no history of myocardial disease. The LV tissue underwent rapid dissection, rapid freezing, and preservation at −80°C when cardiac explants were taken from the operating room. Another LV sample was fixed in either 10% formalin or 2.5% glutaraldehyde.

**Table 1:** Pediatric DCM descriptive data.

**No**	Age^*^ (Year)	**Sex**	**BMI (kg/m^**2**^)**	**BNP (pg/ml)**	**Echocardiography**			**Medications**					**NYHA heart function**
					**LVDD (mm)**	**RVDD (mm)**	**EF %**	**β-blockers**	**Digoxin**	**ACEI**	**Diuretics**	**Aldosterone**	
1	13.4	F	14.98	N	59	54	46	Y	Y	Y	Y	Y	Class IV
2	8.4	M	21.3	7,328	53	42	23	Y	Y	Y	Y	N	Class IV
3	13.9	M	14.27	N	61	50	16	N	Y	Y	N	Y	Class IV
4	12.33	M	13.22	N	53	47	28	Y	Y	Y	Y	Y	Class IV
5	10.67	M	19.63	140.9	60	32	26	Y	Y	Y	N	Y	Class IV
6	9.58	F	18.65	1,994.7	68	32	20	N	Y	Y	N	Y	Class IV
7	13.08	M	U	N	61	39	15	N	N	N	N	N	Class IV
8	11.5	F	U	1,612.5	70	49	12	Y	Y	N	N	Y	Class IV
9	14.83	M	U	5,408.8	74	44	21	Y	N	Y	Y	Y	Class III–IV
10	13.08	F	17.1	N	57	58	25	U	U	U	Y	Y	Class IV
11	16.83	M	16.85	N	60	55	25	N	Y	Y	Y	Y	Class III–IV

Age^*^, age at tissue collection; F, female; M, male; Y, yes; N, no; U, unknown; BMI, body mass index; BNP, brain natriuretic peptide; LVDD, left ventricular end diastolic diameter; RVDD, right ventricular end diastolic diameter; EF, ejection fraction; ACEI, angiotensin converting enzyme inhibitor; NYHA, New York Heart Association.

### Electron Microscopy

Cardiac specimens (2 mm^2^) were fixed with 0.15 M cacodylate buffer at pH 7.4 containing 2 mM calcium chloride at 4°C overnight. Then, 0.15 M cacodylate buffer was used to rinse the specimens 3 times for 10 min each. Next, the specimens were fixed in 1% osmium tetroxide containing 1.5% potassium ferrocyanide in cacodylate buffer for 1 h. We used ultrapure water the wash samples 3 times and stained them en bloc for 1 h. Then, the samples were again washed with ultrapure water. Specimens were dehydrated in a graded acetone series (50, 70, 90, 100%) for 10 min twice. The specimens were fixed and incubated at 60°C for 48 h. We used a diamond trim tool (Daitome Ultra 45°) to trim each block. The slices were placed into 2% uranium acetate saturated alcohol solution and then lead citrate (15 min each staining) and were dried at room temperature overnight. An electron microscope (HITACHI HT7700) was used to observe and collect images.

### qRT-PCR Assay

Total RNA was extracted from LV cardiac tissue by using TRIzol reagent according to the manufacturer's instructions (Sigma, St. Louis, MO), and reverse transcription reactions were performed using an All-in-One synthesis kit (GeneCopoeia, USA) according to the manufacturer's recommendations. RT-PCR was performed using a Bio-Rad CFX manager. A total reaction volume of 10 μl contained 1 μl of cDNA, 5 μl of All-in-One RT-PCR mix solution (GeneCopoeia, USA), 1 μl each of sense and antisense primers (10 μM), and up to 2 μl of ddH_2_O. Primer sequences are listed (Supplement 1). The PCR amplification conditions were 95°C for 10 min and 39 cycles of 95°C for 10 s, 55°C for 30 s, and 72°C for 30 s, followed by 65°C for 5 s and 95°C for 5 s. GAPDH mRNA levels were used for normalization, and the relative mRNA expression was calculated by the 2^−ΔΔ*Ct*^ method.

### Data Analysis

All statistical analyses of qRT-PCR data were performed with GraphPad Prism software (GraphPad Software, Inc.). Variables were compared between the groups using comparison of two groups after analysis of variance (ANOVA). Statistical significance was set a priori at *p* < 0.05, and all data are presented as the mean ± SEM in the figures.

## Results

Children with DCM in this group who underwent heart transplantation had an age range of 8–17 years old, with an average age of 12.5 ± 2.4 years, and the ratio of males to females was 7:4. The LV ejection fraction (LVEF) ranged from 12% to 46%, with an average of 23 ± 9%. The average LV end diastolic diameter (LVDD) was 61.45 ± 6.684 mm, and the average right ventricular end diastolic diameter (RVDD) was 45.64 ± 8.8 mm. The mean BNP level in this group was 3,297 ± 2,967 pg/ml, while the normal value of BNP in our hospital was <100 pg/ml; the BNP value in the DCM group was at least 14 times higher than the normal value. All patients had a New York heart function of IV (Table 1).

### Pathology and Ultrastructure in Pediatric DCM

**Figure 1:**
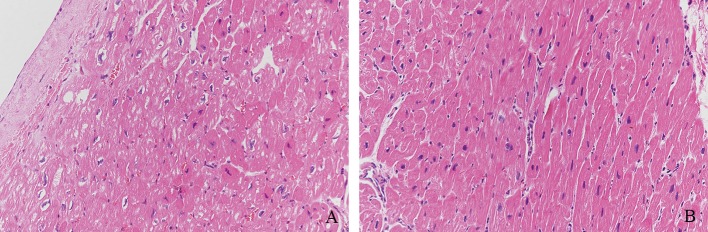
Pathology of HE-stained myocardial tissue. **(A)** LV myocardial fibers of DCM samples showed variable thickness with blurred transverse striae. **(B)** Some myocardial fibers were thick, the nuclei were enlarged and hyperchromatic, and some areas between myocardial tissues were obviously fibrotic (Magnification = 200).

We observed the pathology of HE-stained myocardial tissues by light microscopy. LV myocardial fibers in the DCM group showed a variable thickness with blurred transverse striae. Some myocardial fibers were thick, the nuclei were enlarged and hyperchromatic, and some areas between the myocardial tissues were obviously fibrotic (Figures 1A,B).

**Figure 2:**
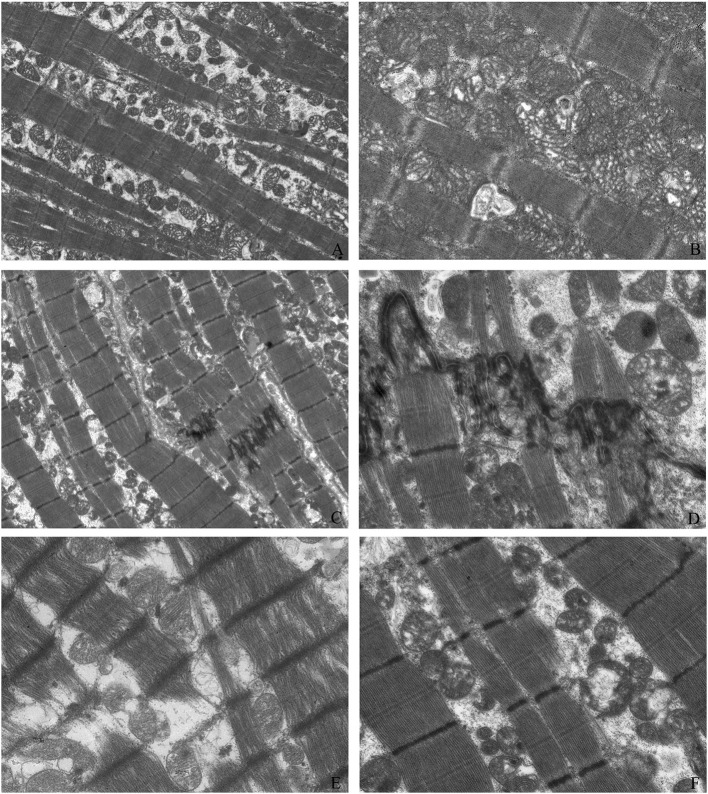
Myocardial tissue ultrastructure. The ultrastructure of control myocardial tissue (**A**: magnification = 2,000, **B**: magnification = 5,000); Z bands of myofibrils from pediatric DCM cardiac tissue appeared enlarged, loose, and fuzzy, and the sarcomeres disappeared (**C**: magnification = 2,000, **D**: magnification = 5,000). Some myofibrils showed dissolution and breakage (**C,E**: magnification = 5,000). Compared with those of the normal group, some mitochondria were swollen and dissolved, and the mitochondrial cristae were empty, blurred, or even absent (**C,F**: magnification = 5,000).

We observed the myocardial tissue ultrastructure in the groups with electron microscopy. Compared with those of the control myocardial tissue, Z bands of myofibrils from the pediatric DCM cardiac tissue appeared enlarged, loose, and fuzzy, and the sarcomeres disappeared. Some myofibrils showed dissolution or breakage. Compared with the normal group, the DCM group did not show an abnormal number of mitochondria, but some mitochondria were swollen and dissolved, and the mitochondrial cristae were empty, blurred or even absent (Figure 2).

### The Gene Expression of Drug-Related Receptors on Cardiomyocytes in Children

**Figure 3:**
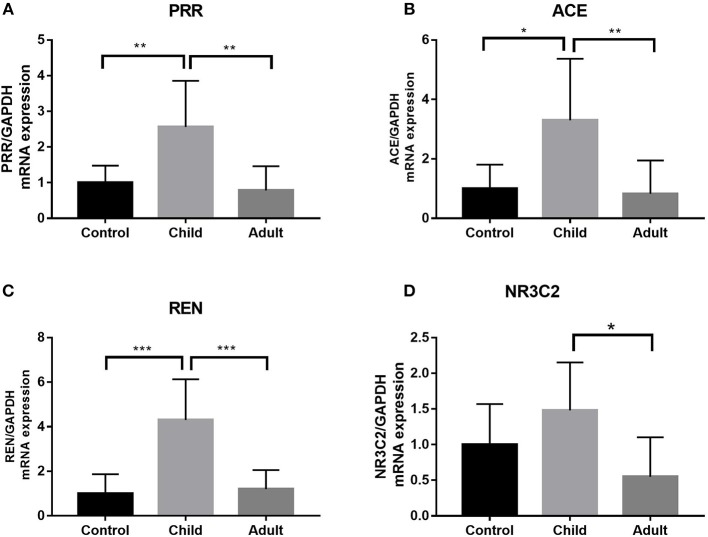
Expression of PRR, ACE, REN, and NR3C2 in pediatric, adult DCM LV samples and control samples. **(A)** PRR expression was higher in pediatric DCM patients than controls. PRR expression was higher in pediatric DCM patients than adults. The significance was determined using comparison of two groups after ANOVA: *p* = 0.009, *p* = 0.0023. **(B)** ACE expression was higher in pediatric DCM patients than controls. ACE expression was higher in pediatric DCM patients than adults. The significance was determined using comparison of two groups after ANOVA: *p* = 0.017, *p* = 0.008. **(C)** REN expression was higher in pediatric DCM patients than controls. REN expression was higher in pediatric DCM patients than adults. The significance was determined using comparison of two groups after ANOVA: *p* = 0.0003, *p* = 0.0006. **(D)** NR3C2 expression was not higher in pediatric DCM patients than controls. Comparison of two groups after ANOVA determined that there was no difference in relative expression. NR3C2 expression was higher in pediatric DCM patients than adults. The significance was determined using comparison of two groups after ANOVA: *p* = 0.015. *P < 0.05, **P < 0.01, ***P < 0.001.

Using qRT-PCR, we analyzed the expression of PRR, REN, ACE, and NR3C2 of the RAAS in the LV tissues from control and pediatric DCM hearts. We found that the expression of PRR, REN, ACE was higher in children with DCM than adults (*p* < 0.05) (Figure 3).

**Figure 4:**
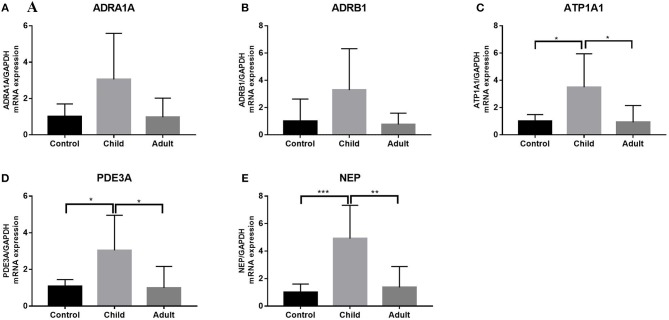
Expression of ATP1A1, ADRA1, ADRB1, PDE3A, and NEP in pediatric, adult DCM LV, and control samples. **(A)** ADRA1 expression was not higher in pediatric DCM patients than controls. ADRA1 expression was not higher in pediatric DCM patients than adults. Comparison of two groups after ANOVA determined that there was no difference in relative expression. **(B)** ADRB1 expression was not higher in pediatric DCM patients than controls. ADRB1 expression was not higher in pediatric DCM patients than adults. Comparison of two groups after ANOVA determined that there was no difference in relative expression. **(C)** ATP1A1 expression was higher in pediatric DCM patients than controls. ATP1A1 expression was higher in pediatric DCM patients than adults. The significance was determined using comparison of two groups after ANOVA: *p* = 0.021, *p* = 0.014. **(D)** PDE3A expression was higher in pediatric DCM patients than controls. PDE3A expression was higher in pediatric DCM patients than adults. The significance was determined using comparison of two groups after ANOVA: *p* = 0.041, *p* = 0.020. **(E)** NEP expression was higher in pediatric DCM patients than controls. NEP expression was higher in pediatric DCM patients than adults. The significance was determined using comparison of two groups after ANOVA: *p* = 0.0008, *p* = 0.0014. *P < 0.05, **P < 0.01, ***P < 0.001.

We measured the expression of ATP1A1, ADRA1, ADRB1, PDE3A, and NEP in the LV tissues from pediatric and normal hearts and found differences (Figure 4). ADRA1 and ADRB1 showed no significant differences between pediatric DCM samples and normal samples (Figures 4A,B). Both ATP1A1 and PDE3A levels were higher in pediatric DCM samples than in normal DCM samples (*p* < 0.05) (Figures 4C,D). NEP expression was significantly elevated in pediatric DCM compared to normal samples (*p* = 0.0008) (Figure 4E).

### Gene Expression of the Drug-Related Receptors on Cardiomyocytes in Adults and Children

Using qRT-PCR, we analyzed the expression of PRR, REN, ACE, and NR3C2 of the RAAS in the LV tissues from adult and pediatric DCM hearts. These gene expression levels were higher in children with DCM than adults (*p* < 0.05) (Figure 3). We also measured the expression of ATP1A1, ADRA1, ADRB1, PDE3A, and NEP in child and adult hearts (Figure 3). ATP1A1, PDE3A, and NEP levels were increased in pediatric DCM and adult samples (*p* < 0.05) (Figures 4C–E). The expression of ADRA1 and ADRB1 was not significantly different between pediatric DCM and adult samples (Figures 4A,B).

## Discussion

The specimens selected in this study were all derived from the myocardial tissues of children with end-stage DCM. Even though the number of pediatric heart transplants has increasing in recent years, the number of pediatric heart transplants is still small relative to that of adult. So, it is not easy to get more samples of DCM heart. But this study firstly analyzed expression levels of drug relevant receptors and myocardial ultrastructure of DCM heart samples. Through electron microscopy, we observed that the end-stage myocardium in these children showed significant degeneration, the myofibrils focally dissolved and disappeared, and the mitochondria showed mitochondrial degeneration and vacuolation. Compared with that of adults, the myocardial ultrastructure in children with end-stage DCM was not significantly different (11).

The most common cause of pediatric HF is DCM, and the clinical prognosis of DCM is poor (12). The increase in the survival rate of these children is largely due to heart transplantation. While the preferred treatment for end-stage HF is transplantation, children on waiting lists for heart transplantation have some of the highest mortality rates because of the limited number of heart donors (13). Therefore, identification of a suitable drug treatment to prolong the survival time before heart transplantation is urgently needed.

Although, the myocellular mechanisms of DCM in children are mostly unexplored, treatment strategies for HF in children are consistent with those in adults. Some clinical studies have found that the treatment of adult patients with HF reduced mortality. However, the same treatment did not improve prognosis in children (14). In general, pharmacotherapy for DCM in children is consistent with pharmacotherapy for DCM in adults. A transition from almost total use of digoxin and diuretics to widespread use of ACE inhibitors occurred in the 1980s (15), and another transition to second- and third-generation β-AR blockers occurred in the 1990s. Milrinone, a PDE3 inhibitor, improves myocardial performance without increasing afterload. Therefore, milrinone can be used to improve cardiac function in children with end-stage HF (16). In addition, LCZ696 was more effective in lowering blood pressure in patients with hypertension and reduced all-cause mortality in patients with HF compared to valsartan or enalapril (17, 18). With the development of research, many researchers have realized that the mechanism of DCM in children and adults may not be the same. We measured the gene expression of the corresponding receptors of drugs used to treat HF on cardiomyocytes, which provides a reference for the treatment of pediatric DCM.

RAAS activation can cause fluid retention, pulmonary venous congestion, pleural effusion, cardiac dilatation, and myocardial fibrosis, leading to clinical symptoms of HF in humans. Active renin exclusively controls the firstrate limiting step in the RAAS cascade, i.e., conversion of angiotensinogen to angiotensin I (19, 20). The PRR binds renin and prorenin and induces profibrotic intracellular signal cascades (21). The NR3C2 gene, which encodes the mineralocorticoid receptor, mediates the effects of aldosterone on salt and water balance within restricted target cells to reduce the load on the heart. In this study, we observed higher expression of PRR, REN, and ACE of the RAAS in the LV tissues from pediatric DCM hearts compared to those of normal and adult hearts. To date, the most commonly used therapy targeting the RAAS system to treat heart disease in children is ACE inhibitors. Our study indicated that ACE inhibitors may block cardiomyocyte expansion and could significantly mitigate HF. In addition, either PRR or REN receptor inhibitors may be effective in DCM. In the future, we will further study the effects of either PRR or REN receptor inhibitors on DCM.

Adult HF patients respond well to treatment with β-blockers because AR-mediated adaptation plays an important role in heart abnormalities in adult HF. However, a growing body of literature suggests that β-blockers are not as effective in treating pediatric HF as adult HF (22). Our results showed that ADRA1 and ADRB1 expression in pediatric DCM was not strikingly different from expression in normal samples. Therefore, we hypothesized that there are different adaptive β-AR and adrenalin signaling pathways in children with DCM compared to adults with DCM. This finding may indicate that β-receptor blockers are not effective in treating pediatric DCM.

Our results showed that ATP1A1 expression in pediatric DCM is higher than expression in normal conditions. The ATP1A1 gene encodes an alpha 1 subunit with good affinity for digoxin. Digoxin is a traditional medicine for the treatment of HF in children. Nakano et al. found that the level of myocardial cAMP in children treated with the PDE3 inhibitor milrinone increased, while the level of cAMP in adults treated with the PDE3 inhibitor remained low and unchanged (23). The results of our study confirmed the conclusions of previous studies and indicated that PDE3 inhibitor treatment is more effective in pediatric DCM than adult DCM.

In recent years, many studies have found that a combination of neprilysin/the RAAS inhibitor sacubitril/valsartan (LCZ696), which provides simultaneous neprilysin inhibition and angiotensin-II receptor blockade, was superior to ACEI/ARB therapy (24, 25). Our results showed that expression of the NEP gene, which encodes neprilysin, is higher than that in normal samples. This finding indicates that LCZ696 may be effective in treating children with DCM.

By comparing the expression of drug receptors related to heart disease in adult, child and normal myocardium, we confirmed that children and adults may not have the same response to DCM treatment in this study. ACE inhibitors, PRR or REN receptor inhibitors, PDE3 inhibitors and LCZ696 may be effective in children with DCM. However, β-receptor blockers are not valid treatments for pediatric DCM. Moreover, high receptor expression was observed in children. These data will improve drug selection for DCM patients, enhance DCM treatment and increase the survival rate.

## Data Availability Statement

All datasets analyzed for this study are included in the manuscript/Supplementary Files.

## Ethics Statement

The studies involving human participants were reviewed and approved by Drug clinical trial ethics committee of Huazhong University of Science and Technology. Written informed consent to participate in this study was provided by the participants' legal guardian/next of kin.

## Author Contributions

JS and HP conception and design of the study. JL and YL performed the statistical analysis. PZ and ND collect sample. QG wrote the first draft of the manuscript. All authors contributed to manuscript revision, read and approved the submitted version.

### Conflict of Interest

The authors declare that the research was conducted in the absence of any commercial or financial relationships that could be construed as a potential conflict of interest.
