# Supplementary material for: Evaluation of Drug-Related Receptors in Children With Dilated Cardiomyopathy
Source: Front Pediatr. 2019 Oct 1;7:387. doi: 10.3389/fped.2019.00387 (PMC6779825; doi:10.3389/fped.2019.00387)
Supplement: Supplementary file 1 [file Table_1.doc]

Supplement 1 The primers used in qRT-PCR

| Primer name | Sequences (5’-3’) |
| --- | --- |
| GAPDH-hF  GAPDH-hR | | TGACTTCAACAGCGACACCCA | | --- | | CACCCTGTTGCTGTAGCCAAA | |
| PRR-hF | AGCTCCGTAATCGCCTGTTTC |
| PRR-hR | GGCTAGATGCTTATGACGAGACA |
| REN-hF  REN-hR | GAACAGAACTCACCCTCCGCTAT  ATGAAGCCCATGCCCACAAC |
| ACE-hF  ACE-hR | TGGATATGGAAACCACCTACAGC  GGTCTTCATATTTCCGGGACG |
| NR3C2-hF  NR3C2-hR | CCAACACCTGAGTTCCTTTCC  GACTACCCCATAATGGCATCC |
| ATP1A1-hF  ATP1A1-hR | GCAGTGTTTCAGGCTAACCAGG  CTCCTTCACGGAACCACAGCA |
| ADRA1-hF  ADRA1-hR | CCTTCTCCGCCATCTTCGAG  GCAGCACAGCACATCCACTG |
| ADRB1-hF  ADRB1-hR | GTGTCCTTCCTGCCCATCCT  CAGCACTTGGGGTCGTTGTA |
| PDE3A-hF  PDE3A-hR | GCAGATCCTTTACTTCATCCTAT  TAAGTTAAGGCCCTGTGAGAA |
| NEP-hF  NEP-hR | AAGAAACAGCGATGGACTC  TTTATGCAGTCTGATGACTTG |
